# Enhanced SARS-CoV-2 neutralization by dimeric IgA

**DOI:** 10.1126/scitranslmed.abf1555

**Published:** 2020-12-07

**Authors:** Zijun Wang, Julio C. C. Lorenzi, Frauke Muecksch, Shlomo Finkin, Charlotte Viant, Christian Gaebler, Melissa Cipolla, Hans-Heinrich Hoffman, Thiago Y. Oliveira, Deena A. Oren, Victor Ramos, Lilian Nogueira, Eleftherios Michailidis, Davide F. Robbiani, Anna Gazumyan, Charles M. Rice, Theodora Hatziioannou, Paul D. Bieniasz, Marina Caskey, Michel C. Nussenzweig

**Affiliations:** 1 Laboratory of Molecular Immunology, The Rockefeller University, New York, NY 10065, USA.; 2 Laboratory of Retrovirology, The Rockefeller University, New York, NY 10065, USA.; 3 Laboratory of Virology and Infectious Disease, The Rockefeller University, New York, NY 10065, USA.; 4 Structural Biology Resource Center, The Rockefeller University, New York, NY 10065, USA.; 5 Institute for Research in Biomedicine, Università della Svizzera italiana, 6500 Bellinzona, Switzerland.; 6 Howard Hughes Medical Institute, The Rockefeller University, New York, NY 10065, USA.

## Abstract

Severe acute respiratory syndrome coronavirus 2 (SARS-CoV-2), the virus that causes coronavirus disease 2019 (COVID-19), primarily infects cells at mucosal surfaces. Serum neutralizing antibody responses are variable and generally low in individuals that suffer mild forms of COVID-19. Although potent immunoglobulin G (IgG) antibodies can neutralize the virus, less is known about secretory antibodies such as IgA that might affect the initial viral spread and transmissibility from the mucosa. Here, we characterize the IgA response to SARS-CoV-2 in a cohort of 149 convalescent individuals after diagnosis with COVID-19. IgA responses in plasma generally correlated with IgG responses. Furthermore, clones of IgM-, IgG-, and IgA-producing B cells were derived from common progenitor cells. Plasma IgA monomers specific to SARS-CoV-2 proteins were demonstrated to be twofold less potent than IgG equivalents. However, IgA dimers, the primary form of antibody in the nasopharynx, were, on average, 15 times more potent than IgA monomers against the same target. Thus, dimeric IgA responses may be particularly valuable for protection against SARS-CoV-2 and for vaccine efficacy.

## Two is better than one for COVID-19

Most individuals that become infected with SARS-CoV-2, the virus that causes COVID-19, produce neutralizing antibodies against the virus. However, the relative contribution of individual antibody isotypes remains unclear. In this study, Wang *et al*. investigated the function of SARS-CoV-2–specific antibodies of the IgA isotype, which exists in both monomeric and dimeric forms. Dimeric IgA antibodies, which are formed by the covalent linkage of two individual IgA monomers, are predominantly found in mucosal tissues, including the upper respiratory tract where SARS-CoV-2 is first encountered. The authors found that dimeric IgA antibodies neutralized SARS-CoV-2 more effectively than their monomeric counterparts. Thus, vaccines that induce dimeric IgA antibodies at mucosal surfaces may be good candidates for protection against SARS-CoV-2.

## Teaser

Human dimeric IgA antibodies are potent neutralizers of SARS-CoV-2.

## INTRODUCTION

Severe acute respiratory syndrome coronavirus 2 (SARS-CoV-2) encodes a trimeric spike surface protein (S) that mediates entry into host cells (*1*, *2*). The virus initially infects epithelial cells in the nasopharynx when the receptor binding domain (RBD) of S interacts with the angiotensin-converting enzyme–2 (ACE-2) receptor (*3*–*6*). SARS-CoV-2 may subsequently spread to other epithelial cells expressing ACE-2 in the lung and gut. These tissues are rich in lymphoid cells that are organized into nasopharynx-associated and gut-associated lymphoid tissues (NALT and GALT, respectively). Vaccines delivered by inhalation to specifically target these tissues appear to be more effective in providing sterilizing protection against SARS-CoV-2 (*7*). Among other specializations, NALT and GALT produce large quantities of immunoglobulin A (IgA) antibodies. These antibodies exist as monomers in circulation, where they make up 15% of the serum antibody pool in healthy individuals. However, IgA is found in higher concentrations in secretions from mucosal surfaces, where it exists predominantly as a dimer covalently linked by J chain (*8*–*10*).

Although most individuals produce antibodies in response to SARS-CoV-2 infection, the neutralizing antibody response is highly variable, with as many as 30% of the population producing antibodies with neutralizing activity below 1:50 in pseudovirus neutralization assays (*11*, *12*). Higher neutralization titers and plasma RBD binding activity are associated with prolonged infection, which is likely due to prolonged exposure to the virus (*11*–*13*). IgG antibody cloning experiments from recovered individuals have revealed that neutralizing antibodies target several distinct and nonoverlapping epitopes on the RBD (*11*, *14*–*18*). Some of these antibodies are potently neutralizing and can prevent or treat infection in animal models (*15*–*19*). Moreover, longitudinal studies indicate that these antibodies may also be protective in humans (*20*–*22*). In a cohort of 113 individuals of varying disease severity, anti-RBD antibody levels and neutralizing activity were predictive of disease outcome (*20*). Individuals that developed higher neutralizing titers earlier ultimately fared better (*20*), as did hospitalized individuals that developed higher anti-spike antibody titers (*21*).

Consistent with the fact that SARS-CoV-2 initially infects in the nasopharynx, IgA antibodies that bind to SARS-CoV-2 are produced rapidly after infection and remain elevated in the plasma for at least 40 days after the onset of symptoms (*23*–*26*). While some viruses, such as influenza virus, show increased susceptibility to dimeric forms of antibodies such as IgA (*27*–*29*), others with lower spike densities that cannot be cross-linked by antibodies, like HIV-1, do not (*30*). IgA antibodies have been shown to bind to the RBD of SARS-CoV-2 and can neutralize the virus (*23*–*25*). However, the precise contribution and molecular nature of the IgA response to SARS-CoV-2 have not been reported to date.

Here, we examined a cohort of 149 convalescent individuals who had confirmed infection with SARS-CoV-2 and their close contacts who had measurable plasma neutralizing activity to investigate the contribution of IgA to anti–SARS-CoV-2 antibody response. The individuals were part of a cohort of SARS-CoV-2–infected people that represent a spectrum of illness severity from mild to hospitalized, all of which survived the infection (*11*). Cloning IgA antibodies from single B cells revealed that the neutralizing activity of monomeric IgA is generally lower than corresponding IgG monomers, but dimeric IgA antibodies are, on average, 15-fold more potent than their monomeric counterparts.

## RESULTS

### Plasma anti–SARS-CoV-2 RBD IgA

**Fig. 1:**
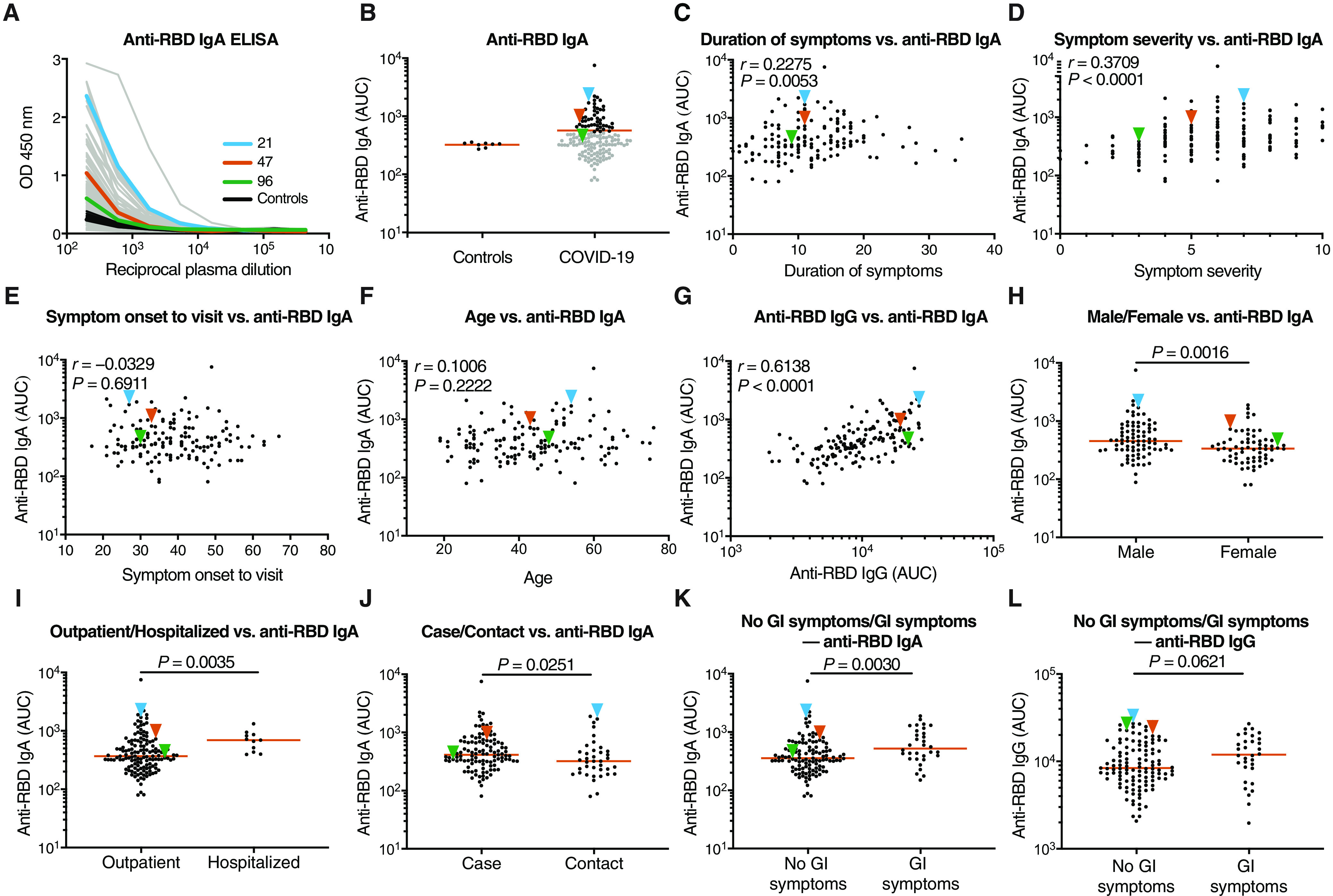
Patients with COVID-19 have plasma IgA antibodies that recognize SARS-CoV-2 RBD. (**A**) ELISAs were used to measure plasma IgA reactivity to the SARS-CoV-2 RBD. The graph shows optical density units at 450 nm (OD) and reciprocal plasma dilutions. Negative controls in black; individuals 21, 47, and 96 in blue, orange, and green lines and arrowheads, respectively (*11*). (**B**) The normalized area under the curve (AUC) values for 8 controls and each of 149 individuals in the cohort were plotted. Horizontal bar indicates mean values. Black dots indicate the individuals that are 2 SDs over the mean of controls, and gray dots represent the individuals below the same parameter. (**C**) The duration of symptoms in days was plotted against normalized AUC for plasma IgA binding to RBD. (**D**) Subjective symptom severity was plotted against the normalized AUC for IgA binding to RBD. (**E**) Symptom onset to time of sample collection in days was plotted against normalized AUC for plasma IgA anti-RBD. (**F**) Age was plotted against normalized AUC for plasma IgA anti-RBD. (**G**) Normalized AUC of plasma anti-RBD IgG ELISA plotted against the normalized AUC for plasma IgA anti-RBD. (**H**) The normalized AUC of anti-RBD IgA ELISA was plotted for males (*n* = 83) and females (*n* = 66). (**I**) The normalized AUC of anti-RBD IgA ELISA was plotted for outpatients (*n* = 138) and hospitalized (*n* = 11) individuals. (**J**) The normalized AUC of plasma anti-RBD IgA ELISA for all cases (*n* = 111) and contacts (*n* = 38) in the cohort was plotted. (**K** and **L**) The normalized AUC of anti-RBD IgA (K) or IgG (L) ELISA for patients with gastrointestinal (GI) symptoms (*n* = 32) and without GI symptoms (*n* = 117) was plotted. The *r* and *P* values for the correlations in (C) to (G) were determined by two-tailed Spearman’s correlations. For (H) to (L), horizontal bars indicate median values. Statistical significance was determined using a two-tailed Mann-Whitney *U* test.

IgM, IgG, and IgA account for 5, 80, and 15% of the antibodies in plasma, respectively. IgG responses to RBD are strongly correlated with neutralizing activity (*11*, *13*–*17*, *31*–*35*). To examine the contribution of IgA to the anti–SARS-CoV-2 RBD response, we tested plasma samples for binding to the RBD by a validated enzyme-linked immunosorbent assay (ELISA). A positive control sample (COV21) was included for normalization of the area under the curve (AUC), and eight independent healthy donor samples were included as negative controls (Fig. 1A). We identified some binding of RBD by IgA and IgM antibodies from healthy donors, similar to that reported for IgG. This binding may reflect some cross-reactivity with seasonal coronaviruses (*11*). Whereas 78 and 15% of the individuals in this cohort showed IgG and IgM anti-RBD concentrations that were at least two SDs above control, only 33% did so for IgA (Fig. 1, A and B) (*11*). Thus, in individuals studied, on average, 40 days after infection, the circulating concentrations of anti-RBD IgA are more modest than IgG and higher than IgM.

Anti-RBD IgA titers were correlated with duration (*P* = 0.005) and severity of symptoms (*P* < 0.0001) but not timing of sample collection relative to onset (*P* = 0.69) or age (*P* = 0.22) (Fig. 1, C to F). Concentrations of anti-RBD IgA antibodies correlated strongly with anti-RBD IgG concentrations (*P* < 0.0001; Fig. 1G). Similar to IgG, females had lower concentrations of RBD-specific IgA than males (*P* = 0.002; Fig. 1H) and hospitalized individuals showed higher anti-RBD IgA titers than those with milder symptoms (*P* = 0.004; Fig. 1I). In addition, cases had higher anti-RBD IgA titers than contacts (*P* = 0.025; Fig. 1J). Individuals that suffered gastrointestinal symptoms showed significantly higher plasma anti-RBD IgA (*P* = 0.003; Fig. 1K) but not IgG titers (*P* = 0.06; Fig. 1L).

### Neutralization activity of purified IgG and IgA

**Fig. 2:**
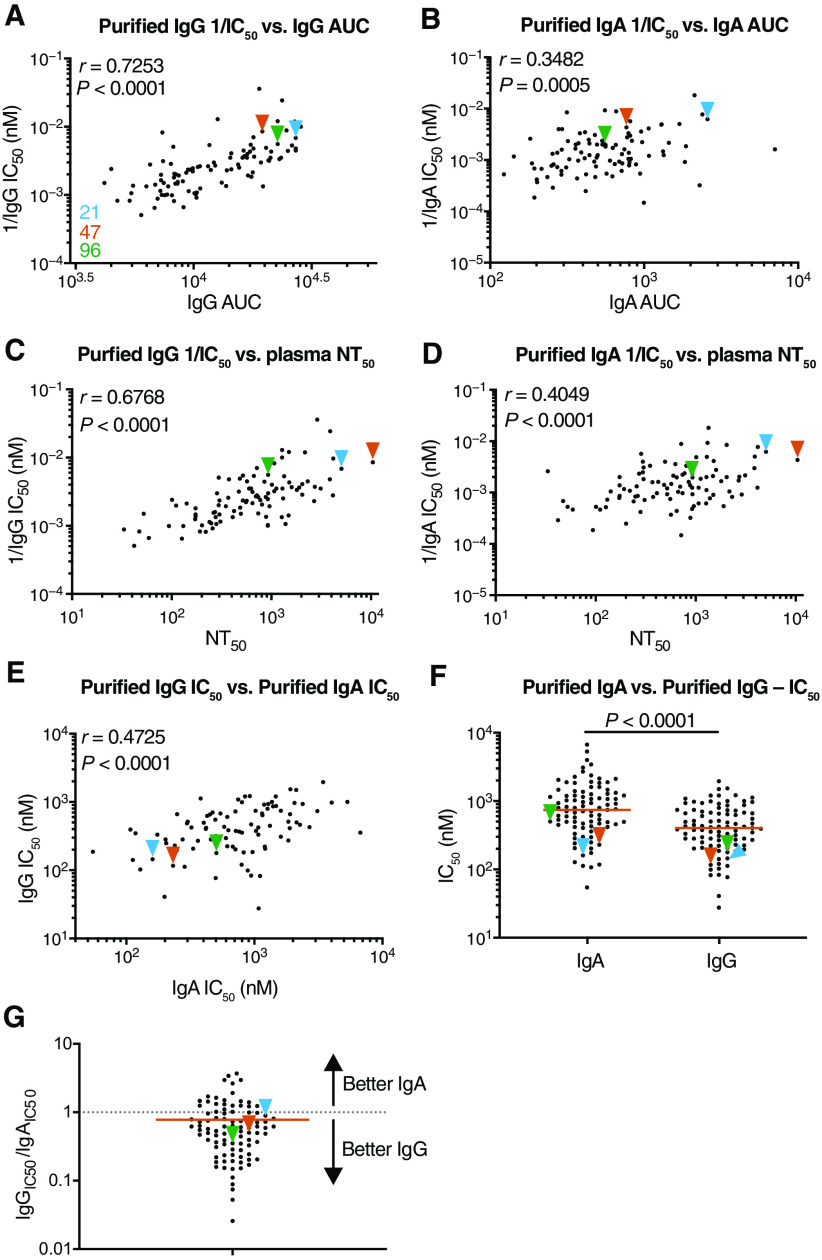
SARS-CoV-2 pseudovirus is neutralized by purified IgA and IgG. Neutralization activity of plasma-purified IgG and IgA from 99 participants was measured in cell lysates of HT1080_ACE2_cl.14 cells 48 hours after infection with pNL4-3ΔEnv-nanoluc–based SARS-CoV-2 pseudovirus. (**A** and **B**) The normalized AUC for plasma IgG (A) or IgA (B) anti-RBD ELISA was plotted against purified IgG (A) or IgA (B) pseudovirus neutralization 1/IC_50_ values. Individuals 21, 47, and 96 are indicated with blue, orange, and green arrowheads, respectively. (**C** and **D**) Published plasma neutralizing titer 50 (NT_50_) values (*11*) were plotted against purified IgG (C) or IgA (D) pseudovirus neutralization 1/IC_50_ values. (**E**) Purified IgA pseudovirus neutralization IC_50_ values were plotted against purified IgG pseudovirus neutralization IC_50_ values. (**F**) Purified IgA and IgG pseudovirus neutralization IC_50_ values were compared. (**G**) The plot depicts the ratio of pseudovirus neutralization IC_50_ values of purified IgG to IgA (*n* = 95). The *r* and *P* values in (A) to (E) and (G) were determined by two-tailed Spearman’s correlations. In (F), *P* values were determined by two-tailed Mann-Whitney *U* tests, and horizontal bars indicate median values. Samples for which purified IgA IC_50_ values could not be detected are not plotted, resulting in *n* = 95 for (B) and (D) to (G).

**Fig. 3:**
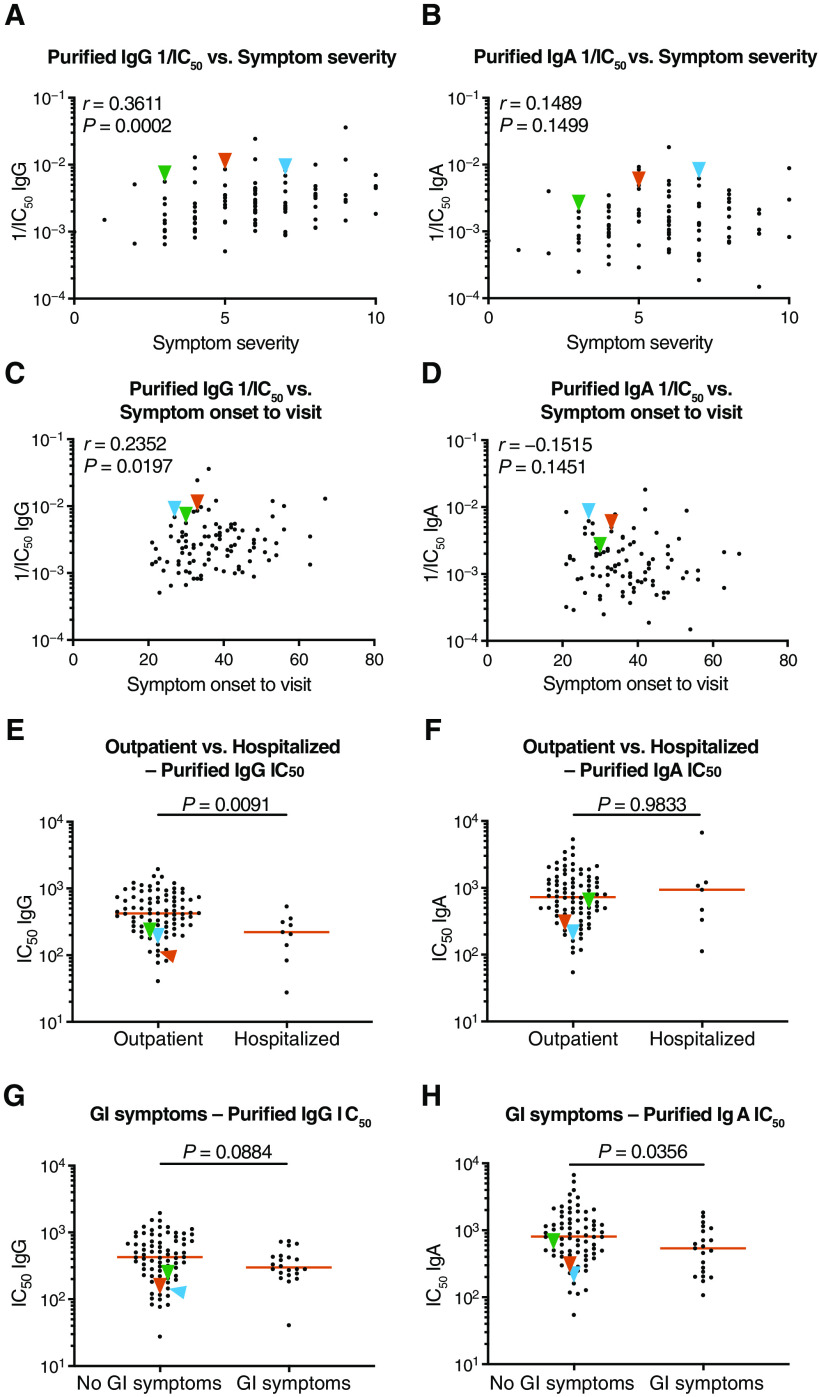
Clinical parameters correlate with plasma IgA or IgG pseudovirus neutralization ability. (**A** and **B**) Symptom severity was plotted against purified IgG (A) and IgA (B) pseudovirus neutralization 1/IC_50_ values. Individuals 21, 47, and 96 are indicated with blue, orange, and green arrowheads, respectively. (**C** and **D**) Symptom onset to time of sample collection in days was plotted against purified plasma IgG (C) and IgA (D) pseudovirus neutralization 1/IC_50_ values. (**E** and **F**) Purified plasma IgG (E) and IgA (F) pseudovirus neutralizing IC_50_ values were compared for all outpatient (*n* = 90) and hospitalized (*n* = 9) participants in the cohort. (**G** and **H**) Purified IgG (G) and IgA (H) pseudovirus neutralization IC_50_ values for patients were compared for patients with GI symptoms (*n* = 21) and without GI symptoms (*n* = 74). The *r* and *P* values in (A) to (D) were determined by two-tailed Spearman’s correlations. In (E) to (H), *P* values were determined by two-tailed Mann-Whitney *U* tests, and horizontal bars indicate median values.

To compare the neutralizing activity of plasma IgA to IgG directly, we purified both isotypes from the plasma of all 99 individuals in our cohort that showed measurable plasma neutralizing activity and tested the two isotypes in an HIV-1–based SARS-CoV-2 pseudovirus neutralization assay (*11*, *34*). Plasma IgG (*P* < 0.0001; Fig. 2A) and IgA (*P* = 0.0005; Fig. 2B) binding to RBD was directly correlated to their neutralizing activity and to the neutralizing activity in plasma (*P* < 0.0001; Fig. 2, C and D, respectively). In addition, there was good correlation between the neutralizing activity of IgG and IgA in a given individual (*P* < 0.0001; Fig. 2E). However, potency of each of the two isotypes varied by as much as two orders of magnitude between individuals (*P* < 0.0001; Fig. 2F). Purified IgG was generally more potent than IgA in neutralizing SARS-CoV-2 pseudovirus in vitro. The geometric mean half-maximal inhibitory concentration (IC_50_) for IgG was 384 nM versus 709 nM for IgA (Fig. 2F). Nevertheless, IgA antibodies were more potent than IgG antibodies in 25% of the individuals tested (Fig. 2G). The two isotypes also differed in that the overall potency of purified IgG was correlated with symptom severity (*P* = 0.0002; Fig. 3A), but purified IgA was not (*P* = 0.15; Fig. 3B). Likewise, potency of purified IgG was correlated with timing of sample collection relative to onset, but purified IgA was not (*P* = 0.020 and *P* = 0.15; Fig. 3, C and D). Neutralizing activities of purified IgG and IgA were not correlated with age, duration of symptoms, or sex (fig. S1). The potency of purified IgG was higher in hospitalized individuals (*P* = 0.009; Fig. 3E), but for IgA, this was not the case (*P* = 0.98; Fig. 3F). Last, the potency of the purified IgA (*P* = 0.036), but not IgG (*P* = 0.09), was greater in individuals that suffered from gastrointestinal symptoms (Fig. 3, G and H).

### Monoclonal anti–SARS-CoV-2 IgM and IgA antibodies

**Fig. 4:**
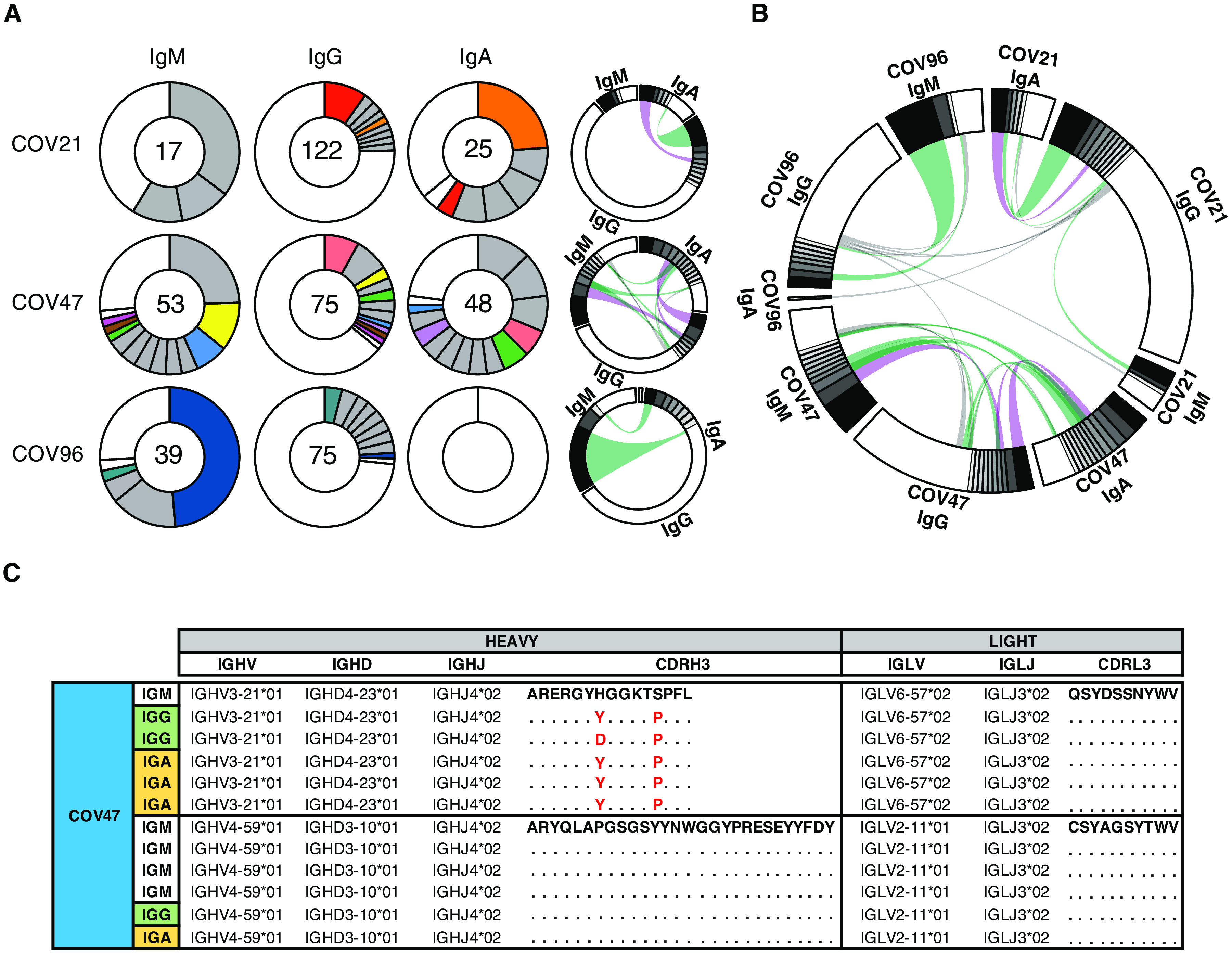
Characterization of monoclonal anti–SARS-CoV-2 RBD IgM, IgG, and IgA antibodies. (**A**) B cells producing IgM, IgG, and IgA from three individuals—COV21, COV47, and COV96—were analyzed, and clonality was evaluated. The number in the inner circle indicates the number of sequences analyzed for the individual denoted. Pie slice size is proportional to the number of clonally related sequences. Colored pie slices indicate clones or singlets that share the same IGHV and IGLV genes and have highly similar CDR3s across isotypes. Gray indicates clones that are not shared. White indicates singlets that are not shared. The right side circos plots show the relationship between antibodies of different isotypes that share the same IGH V(D)J and IGL VJ genes and have highly similar CDR3s. Purple, green, and gray lines connect related clones, clones and singles, and singles to each other, respectively. (**B**) Circos plot shows sequences from all three individuals with clonal relationships depicted as in (A). (**C**) Sample sequence alignment for antibodies of different isotypes isolated from individual COV47 that display the same IGH V(D)J and IGL VJ genes and highly similar CDR3s. Amino acid differences in CDR3s to the reference sequence (bold) are indicated in red, and dots represent identical amino acids.

To characterize the IgM and IgA anti-RBD antibodies elicited by SARS-CoV-2 infection, we used flow cytometry to purify single B lymphocytes that bind to RBD and cloned their antibodies. We obtained 109 IgM- and 74 IgA-matched (64 IgA1 and 10 IgA2) Ig heavy and light chain sequences by reverse transcription and subsequent isotype-specific polymerase chain reaction (PCR) from three convalescent individuals (Fig. 4, A and B). As reported for IgG antibodies (*11*, *14*, *17*, *33*, *36*), the overall number of mutations was generally low when compared to antibodies obtained from individuals suffering from chronic infections such as hepatitis B or HIV-1 (fig. S2) (*37*, *38*). However, the number of V gene nucleotide mutations in IgM and IgA heavy and light chains varied between individuals. For example, in donor COV21, the number of IgM and IgA heavy chain mutations was similar. In contrast, IgM heavy and light chain nucleotide mutations were significantly greater than IgA mutations in COV47 (*P* < 0.0001; fig. S2B). The relatively unexpected similarity between the number of somatic mutation in IgM and IgG could be due to the timing of sample collection early in the immune response before full maturation of the germinal center, wherein most IgG-producing memory cells acquire their mutations (*39*).

Complementarity-determining region 3 (CDR3) length was significantly shorter for IgM than IgA and IgG antibodies (*P* < 0.001; fig. S3), and hydrophobicity was higher for IgM over control but not for IgA and IgG (fig. S4). Compared to the normal human antibody repertoire, several IgA and IgM VH genes were overrepresented, including VH3-53, which can make key contacts with the RBD through germline-encoded CDRH1 and CDRH2 (fig. S5) (*11*, *40*, *41*).

Like IgG antibodies (*11*), IgA and IgM antibodies were found in expanded clones in all three of the individuals examined. Overall, 66.2 and 66.1% of all the IgA and IgM sequences examined were members of expanded clones (Fig. 4, A and B, and data file S1). Nearly identical sequences were shared among the three isotypes in clones found in all three individuals, indicating that switch recombination occurred during B cell clonal expansion in response to SARS-CoV-2 (Fig. 4, B and C). In total, 11 of 55 antigen-specific B cell clones in circulation belonged to expanded clones that contained members expressing different constant regions (Fig. 4C and data files S1 and S2). When compared directly, the neutralizing activity of antibodies that were members of B cell clones producing IgA or IgG varied and did not correlate with one or the other isotype (table S1).

To examine the binding properties of the anti–SARS-CoV-2 monoclonal antibodies, we expressed 46 IgM and 35 IgA antibodies by transient transfection (data file S3). IgM variable regions were produced on an IgG1 backbone to facilitate expression and purification. IgA antibodies were expressed as native IgA1 or IgA2 monomers. ELISAs on RBD showed that 100 and 91.3% of the IgA and IgM antibodies bound to the RBD with an average half-maximal effective concentration of 52.8 and 101.6 ng/ml, respectively (fig. S6, A and B, and data file S4).

**Fig. 5:**
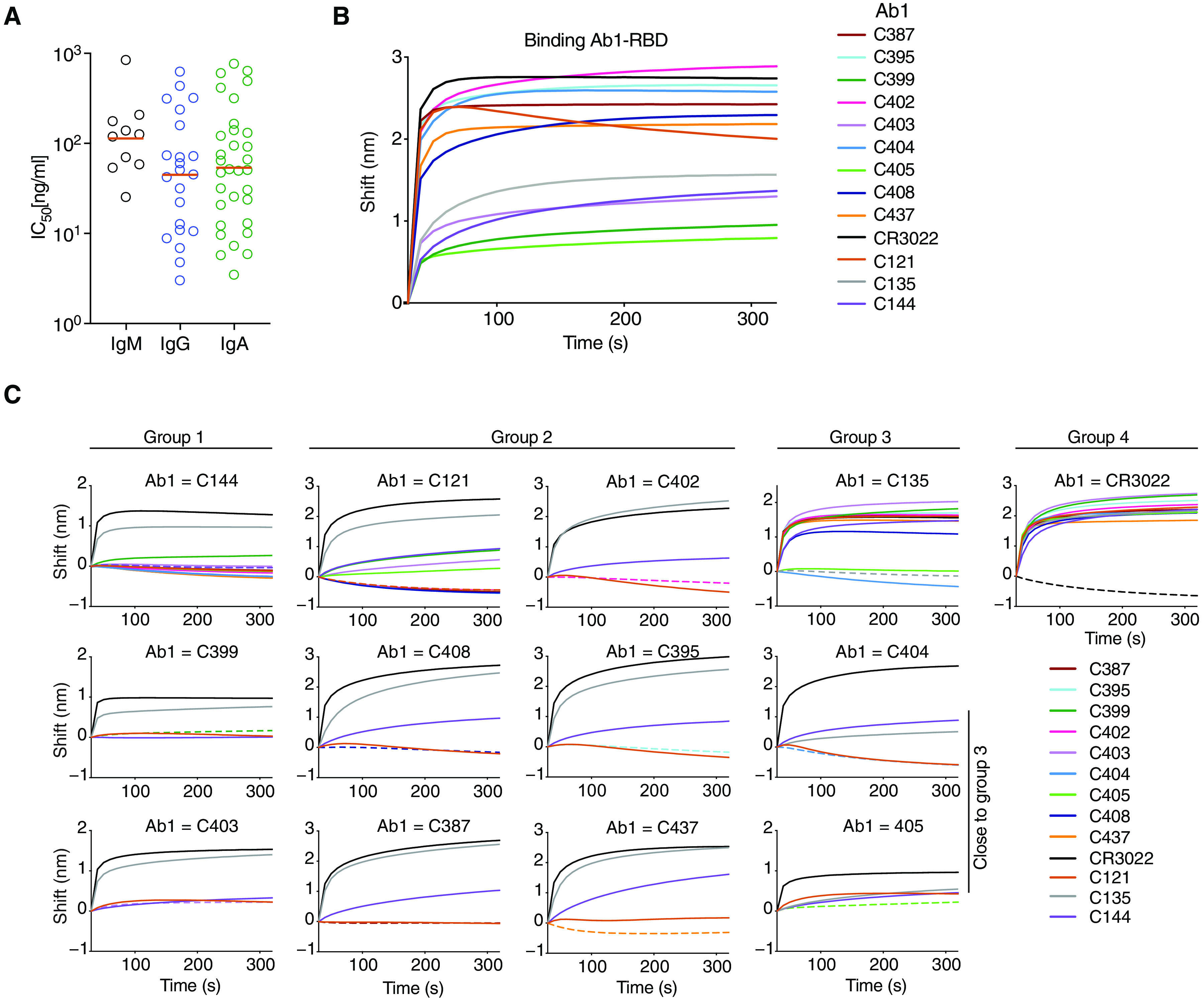
Monoclonal IgA and IgM antibodies bind and neutralize SARS-CoV-2 in vitro. (**A**) Pseudovirus IC_50_ neutralization values for IgA and IgM monoclonal antibodies and IgG monoclonal antibodies from the same individuals (*11*). Antibodies with IC_50_ < 1000 ng/ml are shown. Orange lines indicate geometric mean. (**B**) RBD binding was quantified by biolayer interferometry. (**C**) The binding of a second antibody (Ab2) to preformed first antibody (Ab1)–RBD complexes was also quantified by biolayer interferometry. Dotted line denotes where Ab1 and Ab2 are the same, and Ab2 is color-coded as indicated. We tested selected IgA antibodies against preformed complexes representing antibodies that bind to different structurally determined epitopes (*40*) from class 1, 2, 3, or 4 (C144-, C121-, C135-, or CR3022-RBD, respectively).

To determine neutralizing activity of the IgM and IgA antibodies, we tested them against an HIV-1–based SARS-CoV-2 pseudovirus as either IgG monomers or native IgA monomers, respectively. IgM antibodies were tested as IgG antibodies because of the difficulty in producing IgM pentamers. Among the 42 RBD binding IgM antibodies tested, we found 10 that neutralized the virus in the ng/ml range with an IC_50_ of 114.0 ng/ml (Fig. 5A, fig. S6C, and data file S4). In contrast, 32 of 35 RBD binding IgA antibodies tested neutralized the virus in the ng/ml range with an IC_50_ of 53.6 ng/ml (Fig. 5A, fig. S6C, and data file S4). Thus, IgM antibodies expressed as monomeric IgG antibodies show lower neutralizing activity than either native IgA or IgG monomers (Fig. 5A).

To examine the epitopes targeted by the IgA antibodies with high neutralizing activity, we performed biolayer interferometry experiments in which a preformed antibody-RBD complex consisting of anti-RBD antibodies representing class 1, 2, 3, or 4 as determined by structural analysis (C144-, C121-, C135-, or CR3022-RBD) was exposed to an IgA monoclonal (Fig. 5, B and C) (*11*, *40*, *42*). The IgA monoclonal antibodies bound to RBD with variable affinities (Fig. 5B). Seven of the IgA antibodies were in class 1 or 2 and competed with C144 or C121, and two others competed with C135 and were therefore in class 3 (Fig. 5C and fig. S7).

### Dimeric anti–SARS-CoV-2 IgA is more potent than monomeric IgA

**Fig. 6:**
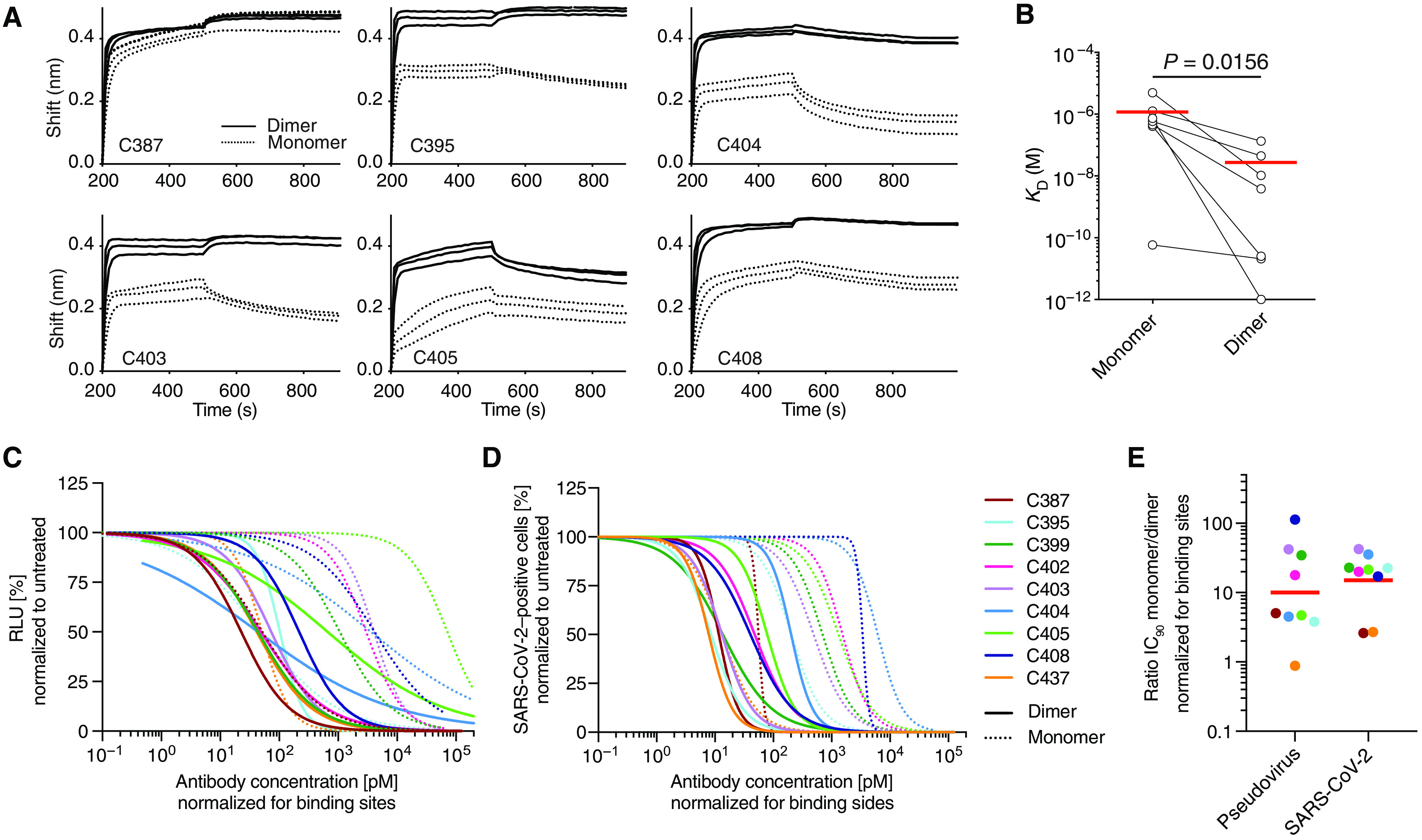
IgA dimers bind to RBD and neutralize authentic SARS-CoV-2 more potently than monomers in vitro. (**A**) Graphs depict binding affinity measurements of individual IgA monomers and corresponding dimers. (**B**) The dissociation constant (*K*_D_) values of monomers and dimers were compared. Horizontal lines indicate mean (*n* = 7). (**C** and **D**) Normalized relative luminescence values (RLU) for cell lysates of 293T_ACE2_ cells after infection with SARS-CoV-2 pseudovirus (C) or normalized percentage of SARS-CoV-2–positive Vero E6 cells 48 hours after infection with SARS-CoV-2 authentic virus (D). Values obtained in the absence of antibody are plotted at *x* = 0.1 to be visible on a log scale in the presence of increasing concentrations of indicated monoclonal antibodies in their monomeric or dimeric form. Four-parameter nonlinear regression curve fits of normalized data are shown. (**E**) IC_90_ values were compared between monomer to dimers after normalization to number of antibody binding sites. For (B), Student’s *t* test was used.

Mucosal IgA exists predominantly as a dimer of two IgA monomers covalently linked together by J chain. To compare the binding properties of IgA monomers and dimers, we coexpressed eight IgA1s and one IgA2 with J chain to produce mixtures of monomers and dimers that were purified by size exclusion chromatography (fig. S8). When tested in biolayer interferometry experiments, the dimers uniformly showed increased apparent affinities compared to the monomers by an average of 43.27-fold (*P* = 0.016; Fig. 6, A and B). To determine whether increased apparent affinity correlates with neutralizing activity, we compared the monomers and dimers in pseudovirus neutralization assays. All but one of the IgA dimers were more potent at neutralizing pseudovirus than the corresponding monomers with differences in activity ranging from 3.8- to 113-fold (Fig. 6C, fig. S9A, and table S2). The relative increase in neutralizing activity between monomer and dimer was inversely correlated with the neutralizing activity of the monomer in this assay (IC_50_: *r* = 0.80, *P* = 0.014; fig. S9B). For example, whereas C437, the most potent antibody, showed equivalent activity as a monomer and dimer, C408, one of the least potent antibodies, was 113-fold more potent as a dimer (fig. S9B).

IgA monomers and dimers were also compared in authentic SARS-CoV-2 microneutralization assays (Fig. 6D and fig. S9, C and D). Neutralizing activities of the nine monomers and nine dimers correlated strongly with those measured in the pseudovirus neutralization assay (IC_50_: *r* = 0.84, *P* < 0.0001; IC_90_: *r* = 0.91, *P* < 0.0001; fig. S9E). On average, there was a 15-fold geometric mean increase in activity for the dimer over the monomer against SARS-CoV-2 and less variability in the degree of enhancement in microneutralization compared to pseudovirus assays (Fig. 6E and table S2). Thus, dimeric IgA is more potent than monomeric IgA against SARS-CoV-2 (Fig. 6E).

## DISCUSSION

Neutralizing antibody titers are the best correlates of protection in most vaccines (*43*). Among antibody isotypes, secretory IgA, which is found at mucosal surfaces, plays a crucial role in protecting against pathogens that target these surfaces (*44*). Serum IgA monomers are produced by the same cells that produce secretory dimers, and we find that serum IgA responses to SARS-CoV-2 correlate with IgG responses. Although the monomeric form of IgA found in serum is, on average, twofold less potent than IgG, the dimeric, secretory form of IgA found in mucosa is more than one log more potent than their respective monomer forms against authentic SARS-CoV-2, suggesting that dimeric IgA is a more potent neutralizer than IgG. The difference in neutralizing activity between the isotypes in serum could be due to differences in the developmental kinetics of the two isotypes during the immune response to this pathogen.

The increased potency of the dimeric form of IgA suggests that cross-linking the S protein on the viral surface enhances neutralizing activity either directly or simply through increased apparent affinity. This observation is consistent with the finding that monovalent Fab fragments of serum IgG antibodies are far less potent than the intact antibody (*40*). In addition, our findings are in agreement with previous reports demonstrating that influenza virus is more susceptible to neutralization by IgA dimers than monomers (*27*–*29*). Whether the effect that we observed in the context of SARS-CoV-2 is due to inter- or intraspike cross-linking is not known, but it indicates that antibodies or drugs designed to block entry by binding to the RBD could be made more potent by increasing their valency.

Limitations of our study include not having tested the native secretory form of IgA in saliva or feces. In addition, we are unable to explain why the monomeric forms of IgG are more potent in neutralizing SARS-CoV-2 than monomeric IgA. We speculate that this might be due to differences in the precise mechanisms of selection for entry into the IgG or IgA memory or plasma cell compartments (*45*). Future studies will be necessary to mechanistically evaluate these differences.

A number of different candidate vaccines to SARS-CoV-2 are currently being evaluated in the clinic, including mucosally delivered vaccines that typically produce more robust mucosal immune responses (*7*). Secretory IgA responses may be particularly important to these efforts in that potent dimeric forms of these antibodies are found at the mucosal surfaces where cells are initially targeted by SARS-CoV-2. Thus, even vaccines that elicit modest neutralizing activity in serum may be protective, because the secretory polymeric forms of antibodies in mucosa can neutralize the virus. Furthermore, vaccines delivered via the mucosal route can elicit superior IgA responses (*46*–*48*). Whether vaccines that are specifically designed to elicit mucosal IgA responses will be particularly effective preventing SARS-CoV-2 infection remains to be determined (*7*).

## MATERIALS AND METHODS

### Study design

The goal of this study was to investigate the IgA response to SARS-CoV-2 in a cohort of 149 convalescent patients after diagnosis of coronavirus disease 2019 (COVID-19). First, we evaluated the overall binding and neutralizing activity of the plasma anti-RBD IgA, IgG, and IgM antibodies. Second, we sequenced and analyzed the B cell receptors (BCRs) of single B cells from peripheral blood and characterized the three isotypes produced by B cells derived from three individual donors. Third, we cloned and expressed monoclonal IgA and IgM antibodies and tested their binding and neutralizing activities. Last, we compared the affinity and neutralization potency of IgA monomers and dimers against SARS-CoV-2 pseudovirus and authentic SARS-CoV-2. Each experiment contained a minimum of two technical replicates.

Samples were obtained from 149 individuals under a study protocol approved by the Rockefeller University in New York from 1 April to 8 May 2020 as described in (*11*). All participants provided written informed consent before participation in the study, and the study was conducted in accordance with Good Clinical Practice and clinical data collection. The study was performed in compliance with all relevant ethical regulations, and the protocol was approved by the Institutional Review Board of the Rockefeller University.

### Purification and quantification of IgA and IgG from plasma

IgA and IgG were purified from samples with measurable neutralizing activity against SARS-CoV-2-RBD (*11*). Plasma (300 μl) was diluted with phosphate-buffered saline (PBS), heat-inactivated (56°C for 1 hour), and incubated with peptide M/agarose (gel-pdm-5; InvivoGen) or protein G/agarose (17-0618-05; GE Healthcare Life Sciences) overnight at 4°C. The suspension was transferred to chromatography columns and washed with 10 column volumes of 1× PBS. IgA and IgG were then eluted with 1.5 ml of 0.1 M glycine (pH 3.0), and pH was immediately adjusted to 7.5 with 1 M tris (pH 8.0). PBS (1×) buffer exchange was achieved using Amicon Ultra Centrifugal Filters (Merck Millipore) through a 30-kDa membrane according to the manufacturer’s instructions. IgA and IgG concentrations were determined by measurement of absorbance at 280 nm using a NanoDrop (Thermo Fisher Scientific) instrument, and samples were stored at 4°C.

### Enzyme-linked immunosorbent assays

ELISAs to evaluate the IgG or IgA binding to SARS-CoV-2 RBD were performed as previously described using a validated assay (*49*, *50*). High binding 96–half-well plates (#3690; Corning) were coated with 50 μl per well of a protein solution (1 μg/ml) in PBS overnight at 4°C. Plates were washed six times with washing buffer containing 1× PBS with 0.05% Tween 20 (Sigma-Aldrich) and incubated with 170 μl of blocking buffer per well containing 1× PBS with 2% bovine serum albumin (BSA) and 0.05% Tween 20 (Sigma-Aldrich) for 1 hour at room temperature. Immediately after blocking, monoclonal antibodies or plasma samples were added in PBS and incubated for 1 hour at room temperature. Plasma samples were assayed at a 1:200 starting dilution and seven additional threefold serial dilutions. Monoclonal antibodies were tested at a starting concentration (10 μg/ml) and 10 additional fourfold serial dilutions. Plates were washed six times with washing buffer and then incubated with anti-human IgG (109-036-088; Jackson ImmunoResearch) or anti-human IgA (A0295; Sigma-Aldrich) secondary antibody conjugated to horseradish peroxidase (HRP) in blocking buffer at 1:5000 or 1:3000 dilution, respectively. Plates were developed by addition of the HRP substrate, 3,3′,5,5″-tetramethylbenzidine (TMB; 34021; Thermo Fisher Scientific), for 10 min (plasma samples) or 4 min (monoclonal antibodies), and then the developing reaction was stopped by adding 50 μl of 1 M H_2_SO_4_. Optical density units were measured at 450 nm in a microplate reader (FLUOStar Omega, BMG Labtech). For plasma samples, a positive control (plasma from patient COV21, diluted 200-fold in PBS) and negative control historical plasma samples were added in duplicate to every assay plate for validation. The average of its signal was used for normalization of all the other values on the same plate with Excel software.

### Cell lines

HT1080_Ace2_ cl.14 cells (*34*), 293T_Ace2_ cells (*11*), and Vero E6 kidney epithelial cells were cultured in Dulbecco’s modified Eagle’s medium supplemented with 10% fetal calf serum at 37°C and 5% CO_2_. In addition, medium for Ace2-overexpressing cell lines contained blasticidin (5 μg/ml), and medium for Vero E6 cells was supplemented with 1% nonessential amino acids. All cell lines have tested negative for contamination with mycoplasma, and parental cell lines were obtained from the American Type Culture Collection.

### Pseudotyped virus neutralization assay

SARS-CoV-2 pseudotyped particles were produced by cotransfection of pSARS-CoV-2 S_trunc_ and pNL4-3ΔEnv-nanoluc in 293T cells (*11*, *34*). Fourfold serially diluted purified plasma IgG/IgA from COVID-19 convalescent individuals and healthy donors or monoclonal antibodies were incubated with the SARS-CoV-2 pseudotyped virus for 1 hour at 37°C. Subsequently, the mixture was incubated with Ace2-expressing cells for 48 hours. HT1080_Ace2_ cl. 14 cells (*34*) were used for plasma-derived IgG or IgA assays, and 293T_Ace2_ cells (*11*) were used for monoclonal antibody assays. After incubation, cells were washed twice with PBS and lysed with Luciferase Cell Culture Lysis 5× Reagent (E1531; Promega). NanoLuc Luciferase activity in lysates was measured using the Nano-Glo Luciferase Assay System (N1150; Promega) with the GloMax Navigator Microplate Luminometer (Promega). Relative luminescence units obtained were normalized to those derived from cells infected with SARS-CoV-2 pseudotyped virus in the absence of plasma-derived or monoclonal antibodies. The half-maximal and 90% inhibitory concentrations for purified plasma IgG or IgA or monoclonal antibodies (IC_50_ and IC_90_) were determined using four-parameter nonlinear regression (GraphPad Prism).

### Antibody sequencing, cloning, and expression

Single B cells were isolated from COV21, COV47, and COV96 patients as previously described (*11*). Briefly, RNA from single cells was reverse-transcribed (18080-044; SuperScript III Reverse Transcriptase, Invitrogen) using random primers (48190011; Invitrogen) and followed by nested PCR amplifications and sequencing using the primers for heavy chain that are listed in data file S5 and primers for light chains from (*51*). Sequence analysis was performed with MacVector. Antibody cloning from PCR products was performed by sequencing and ligation-independent cloning into antibody expression vectors (Igγ1-, IGκ-, IGλ-, Igα1, and Igα2) as detailed in (*52*). The Igα1 and Igα2 vectors were from InvivoGen (pfusess-hcha1 for IgA1 and pfusess-hcha2m1 for IgA2). J chain plasmid was a gift from S. Zolla-Pazner. Recombinant monoclonal antibodies were produced and purified as previously described (*51*, *53*). Briefly, monoclonal antibodies were produced by transient cotransfection of 293-F cells with human heavy chain and light chain antibody expression plasmids using polyethylenimine (catalog no. 408727; Sigma-Aldrich). Seven days after transfection, supernatants were harvested, clarified by centrifugation, and subsequently incubated with peptide M (InvivoGen)/protein G–coupled Sepharose beads (catalog no. gel-pdm-5; InvivoGen; 17-0618-05; GE Healthcare) overnight at 4°C. For dimers, antibodies were produced by transient transfection of Expi293F cells with heavy chain, light chain, and J chain expression plasmids at a 1:1:1 ratio. After 5 days, antibodies were harvested, filtered, and incubated with peptide M overnight and eluted.

### Separation of dimeric IgA from its monomeric form by size exclusion chromatography

A prepacked HiLoad 16/60 Superdex 200 pg (catalog no. 28989335; Cytiva) on the NGC Quest 10 Plus Chromatography System by Bio-Rad was calibrated at room temperature using the HMW Gel Filtration Calibration Kit (catalog no. 28403842; Cytiva) and IgG. After equilibration of the column with PBS, each concentrated IgA preparation was applied onto the column using a 1-ml loop at a flow rate of 0.5 ml/min. Dimers of IgA1 or IgA2 were separated from monomers upon an isocratic elution with 70 ml of PBS. The fractions were pooled, concentrated, and evaluated by SDS–polyacrylamide gel electrophoresis using 4 to 12% bis-tris Novex gels (catalog no. M00652; GenScript) under reducing and nonreducing conditions followed by a Coomassie blue staining (catalog no. ISB1L; Expedeon).

### Microneutralization assay with authentic SARS-CoV-2

Production of SARS-CoV-2 virus and the microneutralization assay were performed as described previously (*11*). Vero E6 cells were seeded at 1 × 10^4^ cells per well into 96-well plates on the day before infection. IgA monomers and dimers were serially diluted (fourfold) in BA-1 medium, consisting of medium 199 (Lonza Inc.) supplemented with 1% BSA and 1× penicillin/streptomycin. The diluted samples were mixed with a constant amount of SARS-CoV-2 and incubated for 1 hour at 37°C. The antibody-virus mix was then directly applied to Vero E6 cells (multiplicity of infection of ~0.1 plaque-forming unit/cell; *n* = 3) and incubated for 22 hours at 37°C. Cells were subsequently fixed by adding an equal volume of 7% formaldehyde to the wells, followed by permeabilization with 0.1% Triton X-100 for 10 min. After extensive washing, cells were incubated for 1 hour at 37°C with blocking solution of 5% goat serum in PBS (catalog no. 005-000-121; Jackson ImmunoResearch). A rabbit polyclonal anti–SARS-CoV-2 nucleocapsid antibody (catalog no. GTX135357; GeneTex) was added to the cells at a 1:1000 dilution in blocking solution and incubated at 4°C overnight. Goat anti-rabbit Alexa Fluor 594 (catalog no. A-11012; Life Technologies) was used as a secondary antibody at a dilution of 1:2000. Nuclei were stained with Hoechst 33342 (catalog no. 62249; Thermo Fisher Scientific) at a 1:1000 dilution. Images were acquired with a fluorescence microscope and analyzed using ImageXpress Micro XLS (Molecular Devices). All experiments involving SARS-CoV-2 were performed in a biosafety level 3 laboratory.

### Biolayer interferometry

Biolayer interferometry assays were performed on the Octet Red instrument (FortéBio) at 30°C with shaking at 1000 rpm. Epitope binding assays were performed with a protein A biosensor (18-5010; FortéBio), following the manufacturer’s protocol “classical sandwich assay.” (i) Sensor check: Sensors immersed 30 s in buffer alone (18-1105; FortéBio). (ii) Capture first Ab: Sensors immersed 10 min with Ab1 at 40 μg/ml. (iii) Baseline: Sensors immersed 30 s in buffer alone. (iv) Blocking: Sensors immersed 5 min with IgG isotype control at 50 μg/ml. (v) Antigen association: Sensors immersed 5 min with RBD at 100 μg/ml. (vi) Baseline: Sensors immersed 30 s in buffer alone. (vii) Association Ab2: Sensors immersed 5 min with Ab2 at 40 μg/ml. Curve fitting was performed using the Octet Data analysis software (FortéBio). Affinity measurement: All measurements of RBD-biot binding to monomer IgA or RBD-biot binding to dimer IgA binding were corrected by subtracting the signal obtained from traces performed with RBD-biot but in the absence of IgA. The kinetic analysis using a high precision streptavidin biosensor (18-5118; FortéBio) was performed as follows. (i) Baseline: 60-s immersion in buffer (kinetics buffer 10×; 18-1105; FortéBio). (ii) Loading: 200-s immersion in a solution with biotinylated RBD at 50 μg/ml. (iii) Baseline: 200-s immersion in buffer. (iv) Association: 300-s immersion in solution with IgA at 100, 50, or 25 μM. (v) Dissociation: 600-s immersion in buffer. Curve fitting was performed using the Octet Data analysis software (FortéBio). Mean dissociation constant (*K*_D_) values were determined by averaging all three binding curves that matched the theoretical fit with *R*^2^ ≥ 0.8.

### Computational analyses of antibody sequences

Antibody sequences were trimmed on the basis of quality and annotated using Igblastn v1.14.0 (*54*) with IMGT domain delineation system. Annotation was performed systematically using Change-O toolkit v.0.4.5 (*55*). Heavy and light chains derived from the same cell were paired, and clonotypes were assigned based on their V and J genes using R and Perl scripts (Zenodo, DOI:10.5281/zenodo.4296189). Nucleotide somatic hypermutation and CDR3 length were determined using in-house R and Perl scripts. For somatic hypermutations, IGHV and IGLV nucleotide sequences were aligned against their closest germlines using Igblastn, and the number of differences was considered nucleotide mutations. The average mutations for V genes were calculated by dividing the sum of all nucleotide mutations across all patients by the number of sequences used for the analysis. Hydrophobicity distribution comparisons were calculated as described in (*11*). The frequency distributions of human V genes in anti–SARS-CoV-2 antibodies from this study were compared to 131,284,220 IgH and IgL sequences generated by Soto *et al*. (*56*) and downloaded from cAb-Rep (*57*), a database of human shared BCR clonotypes available at https://cab-rep.c2b2.columbia.edu/. On the basis of the 81 distinct V genes that make up the 1455 analyzed sequences from Ig repertoire of the three patients present in this study, we selected the IgH and IgL sequences from the database that are partially coded by the same V genes and counted them according to the constant region. The frequencies shown in fig. S5 are relative to the source and isotype analyzed. We used the two-sided binomial test to check whether the number of sequences belonging to a specific IgHV or IgLV gene in the repertoire is different according to the frequency of the same IgV gene in the database. Adjusted *P* values were calculated using the false discovery rate correction.

### Statistical analysis

Statistical analyses were performed with GraphPad Prism 8.0 software. Normally distributed data were analyzed by two-sided *t* test, and skewed data were analyzed by Mann-Whitney test. Comparisons of more than two groups were analyzed by one-way analysis of variance (ANOVA) with correction for multiple comparisons by Dunnett’s method. Correlations were tested by Spearman’s correlation coefficient. Statistical significance was defined as *P* < 0.05 unless stated otherwise. *P* values smaller than 0.05 were considered statistically significant (*P < 0.05, **P < 0.01, ***P < 0.001, and ****P < 0.0001). The data are shown as means and individual data points.

## SUPPLEMENTARY MATERIALS

stm.sciencemag.org/cgi/content/full/13/577/eabf1555/DC1 Fig. S1. Age, duration of symptoms, and sex do not correlate with plasma IgA or IgG neutralization ability. Fig. S2. Analysis of antibody somatic hypermutation. Fig. S3. Analysis of antibody CDR3 length. Fig. S4. Analysis of antibody CDR3 hydrophobicity. Fig. S5. Frequency distributions of human V genes. Fig. S6. Binding and neutralizing activity of anti–SARS-CoV-2 RBD IgA and IgM monomers. Fig. S7. Biolayer interferometry experiment. Fig. S8. Purification of dimeric IgA by size exclusion chromatography. Fig. S9. Neutralizing activity of monoclonal monomeric and dimeric IgA antibodies. Table S1. Inhibitory concentrations of monoclonal antibodies from isotype shared clones. Table S2. Inhibitory concentrations of monoclonal IgA monomers and dimers. Data file S1. Sequences of anti–SARS-CoV-2 antibodies. Data file S2. Sequences of antibodies from isotype shared clones. Data file S3. Sequences of cloned recombinant antibodies. Data file S4. Effective and inhibitory concentrations of monoclonal antibodies. Data file S5. Primers. Data file S6. Raw data for graphs with *n* < 20.

## Appendix

View/request a protocol for this paper from *Bio-protocol*.

## References

[1] A. C. Walls, Y.-J. Park, M. A. Tortorici, A. Wall, A. T. McGuire, D. Veesler, Structure, function, and antigenicity of the SARS-CoV-2 spike glycoprotein. Cell 181, 281–292.e6 (2020). 32155444 10.1016/j.cell.2020.02.058 PMC7102599

[2] P. Zhou, X.-L. Yang, X.-G. Wang, B. Hu, L. Zhang, W. Zhang, H.-R. Si, Y. Zhu, B. Li, C.-L. Huang, H.-D. Chen, J. Chen, Y. Luo, H. Guo, R.-D. Jiang, M.-Q. Liu, Y. Chen, X.-R. Shen, X. Wang, X.-S. Zheng, K. Zhao, Q.-J. Chen, F. Deng, L.-L. Liu, B. Yan, F.-X. Zhan, Y.-Y. Wang, G.-F. Xiao, Z.-L. Shi, A pneumonia outbreak associated with a new coronavirus of probable bat origin. Nature 579, 270–273 (2020). 32015507 10.1038/s41586-020-2012-7 PMC7095418

[3] M. Hoffmann, H. Kleine-Weber, S. Schroeder, N. Krüger, T. Herrler, S. Erichsen, T. S. Schiergens, G. Herrler, N.-H. Wu, A. Nitsche, M. A. Müller, C. Drosten, S. Pöhlmann, SARS-CoV-2 cell entry depends on ACE2 and TMPRSS2 and is blocked by a clinically proven protease inhibitor. Cell 181, 271–280.e8 (2020). 32142651 10.1016/j.cell.2020.02.052 PMC7102627

[4] R. Lu, X. Zhao, J. Li, P. Niu, B. Yang, H. Wu, W. Wang, H. Song, B. Huang, N. Zhu, Y. Bi, X. Ma, F. Zhan, L. Wang, T. Hu, H. Zhou, Z. Hu, W. Zhou, L. Zhao, J. Chen, Y. Meng, J. Wang, Y. Lin, J. Yuan, Z. Xie, J. Ma, W. J. Liu, D. Wang, W. Xu, E. C. Holmes, G. F. Gao, G. Wu, W. Chen, W. Shi, W. Tan, Genomic characterisation and epidemiology of 2019 novel coronavirus: Implications for virus origins and receptor binding. Lancet 395, 565–574 (2020). 32007145 10.1016/S0140-6736(20)30251-8 PMC7159086

[5] D. Wrapp, N. Wang, K. S. Corbett, J. A. Goldsmith, C.-L. Hsieh, O. Abiona, B. S. Graham, J. S. McLellan, Cryo-EM structure of the 2019-nCoV spike in the prefusion conformation. Science 367, 1260–1263 (2020). 32075877 10.1126/science.abb2507 PMC7164637

[6] J. Lan, J. Ge, J. Yu, S. Shan, H. Zhou, S. Fan, Q. Zhang, X. Shi, Q. Wang, L. Zhang, X. Wang, Structure of the SARS-CoV-2 spike receptor-binding domain bound to the ACE2 receptor. Nature 581, 215–220 (2020). 32225176 10.1038/s41586-020-2180-5

[7] A. O. Hassan, N. M. Kafai, I. P. Dmitriev, J. M. Fox, B. K. Smith, I. B. Harvey, R. E. Chen, E. S. Winkler, A. W. Wessel, J. B. Case, E. Kashentseva, B. T. McCune, A. L. Bailey, H. Zhao, L. A. VanBlargan, Y.-N. Dai, M. Ma, L. J. Adams, S. Shrihari, J. E. Danis, L. E. Gralinski, Y. J. Hou, A. Schäfer, A. S. Kim, S. P. Keeler, D. Weiskopf, R. S. Baric, M. J. Holtzman, D. H. Fremont, D. T. Curiel, M. S. Diamond, A single-dose intranasal ChAd vaccine protects upper and lower respiratory tracts against SARS-CoV-2. Cell 183, 169–184.e13 (2020). 32931734 10.1016/j.cell.2020.08.026 PMC7437481

[8] B. J. Underdown, J. M. Schiff, Immunoglobulin A: Strategic defense initiative at the mucosal surface. Annu. Rev. Immunol. 4, 389–417 (1986). 3518747 10.1146/annurev.iy.04.040186.002133

[9] M. E. Koshland, The coming of age of the immunoglobulin J chain. Annu. Rev. Immunol. 3, 425–453 (1985). 2415140 10.1146/annurev.iy.03.040185.002233

[10] O. Pabst, New concepts in the generation and functions of IgA. Nat. Rev. Immunol. 12, 821–832 (2012). 23103985 10.1038/nri3322

[11] D. F. Robbiani, C. Gaebler, F. Muecksch, J. C. C. Lorenzi, Z. Wang, A. Cho, M. Agudelo, C. O. Barnes, A. Gazumyan, S. Finkin, T. Hägglöf, T. Y. Oliveira, C. Viant, A. Hurley, H.-H. Hoffmann, K. G. Millard, R. G. Kost, M. Cipolla, K. Gordon, F. Bianchini, S. T. Chen, V. Ramos, R. Patel, J. Dizon, I. Shimeliovich, P. Mendoza, H. Hartweger, L. Nogueira, M. Pack, J. Horowitz, F. Schmidt, Y. Weisblum, E. Michailidis, A. W. Ashbrook, E. Waltari, J. E. Pak, K. E. Huey-Tubman, N. Koranda, P. R. Hoffman, A. P. West Jr., C. M. Rice, T. Hatziioannou, P. J. Bjorkman, P. D. Bieniasz, M. Caskey, M. C. Nussenzweig, Convergent antibody responses to SARS-CoV-2 in convalescent individuals. Nature 584, 437–442 (2020). 32555388 10.1038/s41586-020-2456-9 PMC7442695

[12] F. Wu, M. Liu, A. Wang, L. Lu, Q. Wang, C. Gu, J. Chen, Y. Wu, S. Xia, Y. Ling, Y. Zhang, J. Xun, R. Zhang, Y. Xie, S. Jiang, T. Zhu, H. Lu, Y. Wen, J. Huang, Evaluating the association of clinical characteristics with neutralizing antibody levels in patients who have recovered from mild COVID-19 in Shanghai, China. JAMA Intern. Med. 180, 1356–1362 (2020). 32808970 10.1001/jamainternmed.2020.4616 PMC9377417

[13] B. Ju, Q. Zhang, J. Ge, R. Wang, J. Sun, X. Ge, J. Yu, S. Shan, B. Zhou, S. Song, X. Tang, J. Yu, J. Lan, J. Yuan, H. Wang, J. Zhao, S. Zhang, Y. Wang, X. Shi, L. Liu, J. Zhao, X. Wang, Z. Zhang, L. Zhang, Human neutralizing antibodies elicited by SARS-CoV-2 infection. Nature 584, 115–119 (2020). 32454513 10.1038/s41586-020-2380-z

[14] P. J. M. Brouwer, T. G. Caniels, K. van der Straten, J. L. Snitselaar, Y. Aldon, S. Bangaru, J. L. Torres, N. M. A. Okba, M. Claireaux, G. Kerster, A. E. H. Bentlage, M. M. van Haaren, D. Guerra, J. A. Burger, E. E. Schermer, K. D. Verheul, N. van der Velde, A. van der Kooi, J. van Schooten, M. J. van Breemen, T. P. L. Bijl, K. Sliepen, A. Aartse, R. Derking, I. Bontjer, N. A. Kootstra, W. J. Wiersinga, G. Vidarsson, B. L. Haagmans, A. B. Ward, G. J. de Bree, R. W. Sanders, M. J. van Gils, Potent neutralizing antibodies from COVID-19 patients define multiple targets of vulnerability. Science 369, 643–650 (2020). 32540902 10.1126/science.abc5902 PMC7299281

[15] L. Liu, P. Wang, M. S. Nair, J. Yu, M. Rapp, Q. Wang, Y. Luo, J. F.-W. Chan, V. Sahi, A. Figueroa, X. V. Guo, G. Cerutti, J. Bimela, J. Gorman, T. Zhou, Z. Chen, K.-Y. Yuen, P. D. Kwong, J. G. Sodroski, M. T. Yin, Z. Sheng, Y. Huang, L. Shapiro, D. D. Ho, Potent neutralizing antibodies against multiple epitopes on SARS-CoV-2 spike. Nature 584, 450–456 (2020). 32698192 10.1038/s41586-020-2571-7

[16] Y. Wu, F. Wang, C. Shen, W. Peng, D. Li, C. Zhao, Z. Li, S. Li, Y. Bi, Y. Yang, Y. Gong, H. Xiao, Z. Fan, S. Tan, G. Wu, W. Tan, X. Lu, C. Fan, Q. Wang, Y. Liu, C. Zhang, J. Qi, G. F. Gao, F. Gao, L. Liu, A noncompeting pair of human neutralizing antibodies block COVID-19 virus binding to its receptor ACE2. Science 368, 1274–1278 (2020). 32404477 10.1126/science.abc2241 PMC7223722

[17] T. F. Rogers, F. Zhao, D. Huang, N. Beutler, A. Burns, W.-t. He, O. Limbo, C. Smith, G. Song, J. Woehl, L. Yang, R. K. Abbott, S. Callaghan, E. Garcia, J. Hurtado, M. Parren, L. Peng, S. Ramirez, J. Ricketts, M. J. Ricciardi, S. A. Rawlings, N. C. Wu, M. Yuan, D. M. Smith, D. Nemazee, J. R. Teijaro, J. E. Voss, I. A. Wilson, R. Andrabi, B. Briney, E. Landais, D. Sok, J. G. Jardine, D. R. Burton, Isolation of potent SARS-CoV-2 neutralizing antibodies and protection from disease in a small animal model. Science 369, 956–963 (2020). 32540903 10.1126/science.abc7520 PMC7299280

[18] S. J. Zost, P. Gilchuk, J. B. Case, E. Binshtein, R. E. Chen, J. P. Nkolola, A. Schäfer, J. X. Reidy, A. Trivette, R. S. Nargi, R. E. Sutton, N. Suryadevara, D. R. Martinez, L. E. Williamson, E. C. Chen, T. Jones, S. Day, L. Myers, A. O. Hassan, N. M. Kafai, E. S. Winkler, J. M. Fox, S. Shrihari, B. K. Mueller, J. Meiler, A. Chandrashekar, N. B. Mercado, J. J. Steinhardt, K. Ren, Y.-M. Loo, N. L. Kallewaard, B. T. McCune, S. P. Keeler, M. J. Holtzman, D. H. Barouch, L. E. Gralinski, R. S. Baric, L. B. Thackray, M. S. Diamond, R. H. Carnahan, J. E. Crowe Jr., Potently neutralizing and protective human antibodies against SARS-CoV-2. Nature 584, 443–449 (2020). 32668443 10.1038/s41586-020-2548-6 PMC7584396

[19] A. Baum, D. Ajithdoss, R. Copin, A. Zhou, K. Lanza, N. Negron, M. Ni, Y. Wei, K. Mohammadi, B. Musser, G. S. Atwal, A. Oyejide, Y. Goez-Gazi, J. Dutton, E. Clemmons, H. M. Staples, C. Bartley, B. Klaffke, K. Alfson, M. Gazi, O. Gonzalez, E. Dick Jr., R. Carrion Jr., L. Pessaint, M. Porto, A. Cook, R. Brown, V. Ali, J. Greenhouse, T. Taylor, H. Andersen, M. G. Lewis, N. Stahl, A. J. Murphy, G. D. Yancopoulos, C. A. Kyratsous, REGN-COV2 antibodies prevent and treat SARS-CoV-2 infection in rhesus macaques and hamsters. Science 370, 1110–1115 (2020). 33037066 10.1126/science.abe2402 PMC7857396

[20] W. F. Garcia-Beltran, E. C. Lam, M. G. Astudillo, D. Yang, T. E. Miller, J. Feldman, B. M. Hauser, T. M. Caradonna, K. L. Clayton, A. D. Nitido, M. R. Murali, G. Alter, R. C. Charles, A. Dighe, J. A. Branda, J. K. Lennerz, D. Lingwood, A. G. Schmidt, A. J. Iafrate, A. B. Balazs, COVID-19 neutralizing antibodies predict disease severity and survival. Cell, 10.1016/j.cell.2020.12.015 , (2020). PMC7837114 33412089

[21] C. Atyeo, S. Fischinger, T. Zohar, M. D. Slein, J. Burke, C. Loos, D. J. McCulloch, K. L. Newman, C. Wolf, J. Yu, K. Shuey, J. Feldman, B. M. Hauser, T. Caradonna, A. G. Schmidt, T. J. Suscovich, C. Linde, Y. Cai, D. Barouch, E. T. Ryan, R. C. Charles, D. Lauffenburger, H. Chu, G. Alter, Distinct early serological signatures track with SARS-CoV-2 survival. Immunity 53, 524–532.e4 (2020). 32783920 10.1016/j.immuni.2020.07.020 PMC7392190

[22] S. F. Lumley, D. O’Donnell, N. E. Stoesser, P. C. Matthews, A. Howarth, S. B. Hatch, B. D. Marsden, S. Cox, T. James, F. Warren, L. J. Peck, T. G. Ritter, Z. de Toledo, L. Warren, D. Axten, R. J. Cornall, E. Y. Jones, D. I. Stuart, G. Screaton, D. Ebner, S. Hoosdally, M. Chand, D. W. Crook, A. M. O’Donnell, C. P. Conlon, K. B. Pouwels, A. S. Walker, T. E. A. Peto, S. Hopkins, T. M. Walker, K. Jeffery, D. W. Eyre;; for the Oxford University Hospitals Staff, Testing Group, Antibody status and incidence of SARS-CoV-2 infection in health care workers.. N. Engl. J. Med. 10.1056/NEJMoa2034545 (2020). 10.1056/NEJMoa2034545 PMC7781098 33369366

[23] H. Ma, W. Zeng, H. He, D. Zhao, D. Jiang, P. Zhou, L. Cheng, Y. Li, X. Ma, T. Jin, Serum IgA, IgM, and IgG responses in COVID-19. Cell. Mol. Immunol. 17, 773–775 (2020). 32467617 10.1038/s41423-020-0474-z PMC7331804

[24] D. Sterlin, A. Mathian, M. Miyara, A. Mohr, F. Anna, L. Claër, P. Quentric, J. Fadlallah, H. Devilliers, P. Ghillani, C. Gunn, R. Hockett, S. Mudumba, A. Guihot, C.-E. Luyt, J. Mayaux, A. Beurton, S. Fourati, T. Bruel, O. Schwartz, J.-M. Lacorte, H. Yssel, C. Parizot, K. Dorgham, P. Charneau, Z. Amoura, G. Gorochov, IgA dominates the early neutralizing antibody response to SARS-CoV-2. Sci. Transl. Med. 13, eabd2223 (2021). 33288662 10.1126/scitranslmed.abd2223 PMC7857408

[25] H.-q. Yu, B.-q. Sun, Z.-f. Fang, J.-c. Zhao, X.-y. Liu, Y.-m. Li, X.-z. Sun, H.-f. Liang, B. Zhong, Z.-f. Huang, P.-y. Zheng, L.-f. Tian, H.-Q. Qu, D.-c. Liu, E.-y. Wang, X.-j. Xiao, S.-y. Li, F. Ye, L. Guan, D.-s. Hu, H. Hakonarson, Z.-g. Liu, N.-s. Zhong, Distinct features of SARS-CoV-2-specific IgA response in COVID-19 patients. Eur. Respir. J. 56, 2001526 (2020). 32398307 10.1183/13993003.01526-2020 PMC7236821

[26] J. Seow, C. Graham, B. Merrick, S. Acors, S. Pickering, K. J. A. Steel, O. Hemmings, A. O’Byrne, N. Kouphou, R. P. Galao, G. Betancor, H. D. Wilson, A. W. Signell, H. Winstone, C. Kerridge, I. Huettner, J. M. Jimenez-Guardeño, M. J. Lista, N. Temperton, L. B. Snell, K. Bisnauthsing, A. Moore, A. Green, L. Martinez, B. Stokes, J. Honey, A. Izquierdo-Barras, G. Arbane, A. Patel, M. K. I. Tan, L. O’Connell, G. O’Hara, E. MacMahon, S. Douthwaite, G. Nebbia, R. Batra, R. Martinez-Nunez, M. Shankar-Hari, J. D. Edgeworth, S. J. D. Neil, M. H. Malim, K. J. Doores, Longitudinal observation and decline of neutralizing antibody responses in the three months following SARS-CoV-2 infection in humans. Nat. Microbiol. 5, 1598–1607 (2020). 33106674 10.1038/s41564-020-00813-8 PMC7610833

[27] T. Suzuki, A. Kawaguchi, A. Ainai, S.-i. Tamura, R. Ito, P. Multihartina, V. Setiawaty, K. N. A. Pangesti, T. Odagiri, M. Tashiro, H. Hasegawa, Relationship of the quaternary structure of human secretory IgA to neutralization of influenza virus. Proc. Natl. Acad. Sci. U.S.A. 112, 7809–7814 (2015). 26056267 10.1073/pnas.1503885112 PMC4485102

[28] Y. Terauchi, K. Sano, A. Ainai, S. Saito, Y. Taga, K. Ogawa-Goto, S.-i. Tamura, T. Odagiri, M. Tashiro, M. Fujieda, T. Suzuki, H. Hasegawa, IgA polymerization contributes to efficient virus neutralization on human upper respiratory mucosa after intranasal inactivated influenza vaccine administration. Hum. Vaccin. Immunother. 14, 1351–1361 (2018). 29425074 10.1080/21645515.2018.1438791 PMC6037454

[29] S. Saito, K. Sano, T. Suzuki, A. Ainai, Y. Taga, T. Ueno, K. Tabata, K. Saito, Y. Wada, Y. Ohara, H. Takeyama, T. Odagiri, T. Kageyama, K. Ogawa-Goto, P. Multihartina, V. Setiawaty, K. N. A. Pangesti, H. Hasegawa, IgA tetramerization improves target breadth but not peak potency of functionality of anti-influenza virus broadly neutralizing antibody. PLOS Pathog. 15, e1007427 (2019). 30605488 10.1371/journal.ppat.1007427 PMC6317788

[30] R. D. Astronomo, S. Santra, L. Ballweber-Fleming, K. G. Westerberg, L. Mach, T. Hensley-McBain, L. Sutherland, B. Mildenberg, G. Morton, N. L. Yates, G. J. Mize, J. Pollara, F. Hladik, C. Ochsenbauer, T. N. Denny, R. Warrier, S. Rerks-Ngarm, P. Pitisuttithum, S. Nitayapan, J. Kaewkungwal, G. Ferrari, G. M. Shaw, S.-M. Xia, H.-X. Liao, D. C. Montefiori, G. D. Tomaras, B. F. Haynes, J. M. McElrath, Neutralization takes precedence over IgG or IgA isotype-related functions in mucosal HIV-1 antibody-mediated protection. EBioMedicine 14, 97–111 (2016). 27919754 10.1016/j.ebiom.2016.11.024 PMC5161443

[31] X. Chi, R. Yan, J. Zhang, G. Zhang, Y. Zhang, M. Hao, Z. Zhang, P. Fan, Y. Dong, Y. Yang, Z. Chen, Y. Guo, J. Zhang, Y. Li, X. Song, Y. Chen, L. Xia, L. Fu, L. Hou, J. Xu, C. Yu, J. Li, Q. Zhou, W. Chen, A neutralizing human antibody binds to the N-terminal domain of the Spike protein of SARS-CoV-2. Science 369, 650–655 (2020). 32571838 10.1126/science.abc6952 PMC7319273

[32] Y. Cao, B. Su, X. Guo, W. Sun, Y. Deng, L. Bao, Q. Zhu, X. Zhang, Y. Zheng, C. Geng, X. Chai, R. He, X. Li, Q. Lv, H. Zhu, W. Deng, Y. Xu, Y. Wang, L. Qiao, Y. Tan, L. Song, G. Wang, X. Du, N. Gao, J. Liu, J. Xiao, X.-d. Su, Z. Du, Y. Feng, C. Qin, C. Qin, R. Jin, X. S. Xie, Potent neutralizing antibodies against SARS-CoV-2 identified by high-throughput single-cell sequencing of convalescent patients’ B cells. Cell 182, 73–84.e16 (2020). 32425270 10.1016/j.cell.2020.05.025 PMC7231725

[33] C. Kreer, M. Zehner, T. Weber, M. S. Ercanoglu, L. Gieselmann, C. Rohde, S. Halwe, M. Korenkov, P. Schommers, K. Vanshylla, V. Di Cristanziano, H. Janicki, R. Brinker, A. Ashurov, V. Krähling, A. Kupke, H. Cohen-Dvashi, M. Koch, J. M. Eckert, S. Lederer, N. Pfeifer, T. Wolf, M. J. G. T. Vehreschild, C. Wendtner, R. Diskin, H. Gruell, S. Becker, F. Klein, Longitudinal isolation of potent near-germline SARS-CoV-2-neutralizing antibodies from COVID-19 patients. Cell 182, 843–854.e12 (2020). 32673567 10.1016/j.cell.2020.06.044 PMC7355337

[34] F. Schmidt, Y. Weisblum, F. Muecksch, H.-H. Hoffmann, E. Michailidis, J. C. C. Lorenzi, P. Mendoza, M. Rutkowska, E. Bednarski, C. Gaebler, M. Agudelo, A. Cho, Z. Wang, A. Gazumyan, M. Cipolla, M. Caskey, D. F. Robbiani, M. C. Nussenzweig, C. M. Rice, T. Hatziioannou, P. D. Bieniasz, Measuring SARS-CoV-2 neutralizing antibody activity using pseudotyped and chimeric viruses. J. Exp. Med. 217, e20201181 (2020). 32692348 10.1084/jem.20201181 PMC7372514

[35] R. Shi, C. Shan, X. Duan, Z. Chen, P. Liu, J. Song, T. Song, X. Bi, C. Han, L. Wu, G. Gao, X. Hu, Y. Zhang, Z. Tong, W. Huang, W. J. Liu, G. Wu, B. Zhang, L. Wang, J. Qi, H. Feng, F.-S. Wang, Q. Wang, G. F. Gao, Z. Yuan, J. Yan, A human neutralizing antibody targets the receptor-binding site of SARS-CoV-2. Nature 584, 120–124 (2020). 32454512 10.1038/s41586-020-2381-y

[36] E. Seydoux, L. J. Homad, A. J. MacCamy, K. R. Parks, N. K. Hurlburt, M. F. Jennewein, N. R. Akins, A. B. Stuart, Y.-H. Wan, J. Feng, R. E. Whaley, S. Singh, M. Boeckh, K. W. Cohen, M. J. McElrath, J. A. Englund, H. Y. Chu, M. Pancera, A. T. McGuire, L. Stamatatos, Analysis of a SARS-CoV-2-infected individual reveals development of potent neutralizing antibodies with limited somatic mutation. Immunity 53, 98–105.e5 (2020). 32561270 10.1016/j.immuni.2020.06.001 PMC7276322

[37] J. F. Scheid, H. Mouquet, N. Feldhahn, M. S. Seaman, K. Velinzon, J. Pietzsch, R. G. Ott, R. M. Anthony, H. Zebroski, A. Hurley, A. Phogat, B. Chakrabarti, Y. Li, M. Connors, F. Pereyra, B. D. Walker, H. Wardemann, D. Ho, R. T. Wyatt, J. R. Mascola, J. V. Ravetch, M. C. Nussenzweig, Broad diversity of neutralizing antibodies isolated from memory B cells in HIV-infected individuals. Nature 458, 636–640 (2009). 19287373 10.1038/nature07930

[38] Q. Wang, E. Michailidis, Y. Yu, Z. Wang, A. M. Hurley, D. A. Oren, C. T. Mayer, A. Gazumyan, Z. Liu, Y. Zhou, T. Schoofs, K.-h. Yao, J. P. Nieke, J. Wu, Q. Jiang, C. Zou, M. Kabbani, C. Quirk, T. Oliveira, K. Chhosphel, Q. Zhang, W. M. Schneider, C. Jahan, T. Ying, J. Horowitz, M. Caskey, M. Jankovic, D. F. Robbiani, Y. Wen, Y. P. de Jong, C. M. Rice, M. C. Nussenzweig, A combination of human broadly neutralizing antibodies against hepatitis B virus HBsAg with distinct epitopes suppresses escape mutations. Cell Host Microbe 28, 335–349.e6 (2020). 32504577 10.1016/j.chom.2020.05.010 PMC8182833

[39] G. D. Victora, M. C. Nussenzweig, Germinal centers. Annu. Rev. Immunol. 30, 429–457 (2012). 22224772 10.1146/annurev-immunol-020711-075032

[40] C. O. Barnes, A. P. West Jr., K. E. Huey-Tubman, M. A. G. Hoffmann, N. G. Sharaf, P. R. Hoffman, N. Koranda, H. B. Gristick, C. Gaebler, F. Muecksch, J. C. C. Lorenzi, S. Finkin, T. Hägglöf, A. Hurley, K. G. Millard, Y. Weisblum, F. Schmidt, T. Hatziioannou, P. D. Bieniasz, M. Caskey, D. F. Robbiani, M. C. Nussenzweig, P. J. Bjorkman, Structures of human antibodies bound to SARS-CoV-2 spike reveal common epitopes and recurrent features of antibodies. Cell 182, 828–842.e16 (2020). 32645326 10.1016/j.cell.2020.06.025 PMC7311918

[41] M. Yuan, H. Liu, N. C. Wu, C.-C. D. Lee, X. Zhu, F. Zhao, D. Huang, W. Yu, Y. Hua, H. Tien, T. F. Rogers, E. Landais, D. Sok, J. G. Jardine, D. R. Burton, I. A. Wilson, Structural basis of a shared antibody response to SARS-CoV-2. Science 369, 1119–1123 (2020). 32661058 10.1126/science.abd2321 PMC7402627

[42] M. Yuan, N. C. Wu, X. Zhu, C.-C. D. Lee, R. T. Y. So, H. Lv, C. K. P. Mok, I. A. Wilson, A highly conserved cryptic epitope in the receptor binding domains of SARS-CoV-2 and SARS-CoV. Science 368, 630–633 (2020). 32245784 10.1126/science.abb7269 PMC7164391

[43] S. A. Plotkin, Correlates of protection induced by vaccination. Clin. Vaccine Immunol. 17, 1055–1065 (2010). 20463105 10.1128/CVI.00131-10 PMC2897268

[44] K. Chen, G. Magri, E. K. Grasset, A. Cerutti, Rethinking mucosal antibody responses: IgM, IgG and IgD join IgA. Nat. Rev. Immunol. 20, 427–441 (2020). 32015473 10.1038/s41577-019-0261-1 PMC10262260

[45] C. Viant, G. H. J. Weymar, A. Escolano, S. Chen, H. Hartweger, M. Cipolla, A. Gazumyan, M. C. Nussenzweig, Antibody affinity shapes the choice between memory and germinal center B cell fates. Cell 183, 1298–1311.e11 (2020). 33125897 10.1016/j.cell.2020.09.063 PMC7722471

[46] K. Eriksson, M. Quiding-Järbrink, J. Osek, Å. Möller, S. Björk, J. Holmgren, C. Czerkinsky, Specific-antibody-secreting cells in the rectums and genital tracts of nonhuman primates following vaccination. Infect. Immun. 66, 5889–5896 (1998). 9826370 10.1128/iai.66.12.5889-5896.1998 PMC108746

[47] A. Kantele, M. Häkkinen, Z. Moldoveanu, A. Lu, E. Savilahti, R. D. Alvarez, S. Michalek, J. Mestecky, Differences in immune responses induced by oral and rectal immunizations with *Salmonella typhi* Ty21a: Evidence for compartmentalization within the common mucosal immune system in humans. Infect. Immun. 66, 5630–5635 (1998). 9826335 10.1128/iai.66.12.5630-5635.1998 PMC108711

[48] S. Hopkins, J. P. Kraehenbuhl, F. Schödel, A. Potts, D. Peterson, P. de Grandi, D. Nardelli-Haefliger, A recombinant Salmonella typhimurium vaccine induces local immunity by four different routes of immunization. Infect. Immun. 63, 3279–3286 (1995). 7642256 10.1128/iai.63.9.3279-3286.1995 PMC173452

[49] A. Grifoni, D. Weiskopf, S. I. Ramirez, J. Mateus, J. M. Dan, C. R. Moderbacher, S. A. Rawlings, A. Sutherland, L. Premkumar, R. S. Jadi, D. Marrama, A. M. de Silva, A. Frazier, A. F. Carlin, J. A. Greenbaum, B. Peters, F. Krammer, D. M. Smith, S. Crotty, A. Sette, Targets of T cell responses to SARS-CoV-2 coronavirus in humans with COVID-19 disease and unexposed individuals. Cell 181, 1489–1501.e15 (2020). 32473127 10.1016/j.cell.2020.05.015 PMC7237901

[50] F. Amanat, D. Stadlbauer, S. Strohmeier, T. H. O. Nguyen, V. Chromikova, M. McMahon, K. Jiang, G. A. Arunkumar, D. Jurczyszak, J. Polanco, M. Bermudez-Gonzalez, G. Kleiner, T. Aydillo, L. Miorin, D. S. Fierer, L. A. Lugo, E. M. Kojic, J. Stoever, S. T. H. Liu, C. Cunningham-Rundles, P. L. Felgner, T. Moran, A. García-Sastre, D. Caplivski, A. C. Cheng, K. Kedzierska, O. Vapalahti, J. M. Hepojoki, V. Simon, F. Krammer, A serological assay to detect SARS-CoV-2 seroconversion in humans. Nat. Med. 26, 1033–1036 (2020). 32398876 10.1038/s41591-020-0913-5 PMC8183627

[51] T. Tiller, E. Meffre, S. Yurasov, M. Tsuiji, M. C. Nussenzweig, H. Wardemann, Efficient generation of monoclonal antibodies from single human B cells by single cell RT-PCR and expression vector cloning. J. Immunol. Methods 329, 112–124 (2008). 17996249 10.1016/j.jim.2007.09.017 PMC2243222

[52] L. von Boehmer, C. Liu, S. Ackerman, A. D. Gitlin, Q. Wang, A. Gazumyan, M. C. Nussenzweig, Sequencing and cloning of antigen-specific antibodies from mouse memory B cells. Nat. Protoc. 11, 1908–1923 (2016). 27658009 10.1038/nprot.2016.102

[53] D. F. Robbiani, L. Bozzacco, J. R. Keeffe, R. Khouri, P. C. Olsen, A. Gazumyan, D. Schaefer-Babajew, S. Avila-Rios, L. Nogueira, R. Patel, S. A. Azzopardi, L. F. K. Uhl, M. Saeed, E. E. Sevilla-Reyes, M. Agudelo, K.-H. Yao, J. Golijanin, H. B. Gristick, Y. E. Lee, A. Hurley, M. Caskey, J. Pai, T. Oliveira, E. A. Wunder Jr., G. Sacramento, N. Nery Jr., C. Orge, F. Costa, M. G. Reis, N. M. Thomas, T. Eisenreich, D. M. Weinberger, A. R. P. de Almeida, A. P. West Jr., C. M. Rice, P. J. Bjorkman, G. Reyes-Teran, A. I. Ko, M. R. MacDonald, M. C. Nussenzweig, Recurrent potent human neutralizing antibodies to Zika virus in Brazil and Mexico. Cell 169, 597–609.e11 (2017). 28475892 10.1016/j.cell.2017.04.024 PMC5492969

[54] J. Ye, N. Ma, T. L. Madden, J. M. Ostell, IgBLAST: An immunoglobulin variable domain sequence analysis tool. Nucleic Acids Res. 41, W34–W40 (2013). 23671333 10.1093/nar/gkt382 PMC3692102

[55] N. T. Gupta, J. A. Vander Heiden, M. Uduman, D. Gadala-Maria, G. Yaari, S. H. Kleinstein, Change-O: A toolkit for analyzing large-scale B cell immunoglobulin repertoire sequencing data. Bioinformatics 31, 3356–3358 (2015). 26069265 10.1093/bioinformatics/btv359 PMC4793929

[56] C. Soto, R. G. Bombardi, A. Branchizio, N. Kose, P. Matta, A. M. Sevy, R. S. Sinkovits, P. Gilchuk, J. A. Finn, J. E. Crowe Jr., High frequency of shared clonotypes in human B cell receptor repertoires. Nature 566, 398–402 (2019). 30760926 10.1038/s41586-019-0934-8 PMC6949180

[57] Y. Guo, K. Chen, P. D. Kwong, L. Shapiro, Z. Sheng, cAb-Rep: A database of curated antibody repertoires for exploring antibody diversity and predicting antibody prevalence. Front. Immunol. 10, 2365 (2019). 31649674 10.3389/fimmu.2019.02365 PMC6794461

